# Kritische Lebensereignisse und die Aufnahme freiwilligen Engagements bei Erwachsenen

**DOI:** 10.1007/s40955-023-00241-1

**Published:** 2023-05-12

**Authors:** Richard Benthin

**Affiliations:** Freie Universität Berlin, Berlin, Deutschland

**Keywords:** Kritische Ereignisse, Ehrenamt, Bewältigung, Motive, Transformation, Qualitative Typenbildung, Critical incidents, Volunteering, Coping, Motives, Transformation, Qualitative typing

## Abstract

Freiwilliges Engagement ist eine zentrale Form gesellschaftlicher Teilhabe und bildet einen wichtigen Erfahrungsraum für lebenslanges Lernen. Welche Rolle freiwilliges Engagement als potenzieller Lernraum für die Bewältigung kritischer Lebensereignisse von Ehrenamtlichen spielt, ist jedoch bislang nicht untersucht. Eine solche Betrachtung könnte dazu beitragen, die Genese bislang weitgehend isoliert untersuchter Motive biografisch einzubetten und somit ein tieferes Verständnis für die Grundlagen freiwilligen Engagements zu erlangen. Gegenstand des vorliegenden Artikels ist die motivationstheoretisch und transformationstheoretisch fundierte empirische Untersuchung des Verhältnisses zwischen kritischen Lebensereignissen und der Aufnahme eines ehrenamtlichen Engagements. Auf der Basis einer explorativen, qualitativen Inhaltsanalyse von neun episodischen Interviews werden drei Typen der Bewältigung kritischer Ereignisse durch freiwilliges Engagement vorgestellt. Abschließend werden die präsentierten Typen unter Bezugnahme auf bestehende empirische Befunde diskutiert.

## Abstract (en)

Volunteering is a central way of societal participation and provides and important opportunity of lifelong learning. However, the role of volunteering for coping with critical life incidents as a potential learning space remain not investigated so far. By the biographical embedding of so far isolated motives such a consideration could lead to a deeper understanding of the basics to start volunteering. Referring to motivational and transformative theories, I empirically examine the relationship between critical incidents and the decision for volunteering. Based on an explorative, qualitative content analysis of nine episodic interviews I identify three different types of coping with critical life incidents through volunteering. Finally, results will be discussed referring to existing evidence.

## Einleitung

Freiwilliges Engagement ist in der Erwachsenenbildung bereits seit einiger Zeit als Erfahrungsraum informeller Lern- und Bildungsprozesse etabliert. Es fördert politische Bildung etwa im Engagementbereich von Flucht und Migration (Sprung und Kukovetz 2018) und kann als temporärer Ort des Autonomie‑, Anerkennungs- sowie Authentizitätserlebens für Engagierte verstanden werden (Benedetti 2015). Sowohl qualitative (Rüber et al. 2018) als auch quantitative (Rüber und Janmaat 2021) Untersuchungen konstatieren jüngst ein positives bzw. zum Teil ein reziprokes (Sung et al. 2023) Verhältnis zwischen der Teilnahme an Erwachsenenbildungsangeboten und der Aufnahme eines freiwilligen Engagements. In diesem erlangen Engagierte positive Erträge für ihre physische und psychische Gesundheit sowie für den Aufbau sozialen Kapitals (Wilson 2012) durch gesteigertes Selbstwirksamkeitserleben (Son und Wilson 2017), durch das Erlernen neuer Kompetenzen, durch die Umsetzung individueller Werte und Einstellungen sowie durch die Erweiterung sozialer Netzwerke (Simonson et al. 2017). Weitere Arbeiten betrachten bereits Veränderungen in der Bereitschaft zu freiwilligem Engagement in Bezug auf Übergänge und Ereignisse im Lebenslauf (Lancee und Radl 2014). Jedoch verbleiben die Fragen nach spezifischen biografischen Dispositionen für die Aufnahme und Aufrechterhaltung eines freiwilligen Engagements sowie nach konkreten Lernprozessen, -formen und -inhalten im Kontext biografischer Hintergründe derzeit offen.

Besondere Relevanz gewinnen diese Fragen vor dem Hintergrund der freiwilligen Teilnahme an informellen Lernprozessen, wie etwa im freiwilligen Engagement, wodurch lebensgeschichtliche Ereignisse eine wesentliche Größe für das Verständnis von Motivstrukturen freiwillig Engagierter werden (Nittel 2018). Obwohl die durch die pandemische Ausbreitung von COVID-19 ausgelöste gesellschaftliche Krise in der Bevölkerung eine enorme Engagementbereitschaft auslöste und seit 2020 eine Reihe von Studien die Auswirkungen der COVID-19-Pandemie auf die Erwachsenenbildung untersuchte, bleibt die Rolle des informellen Lernens in der Pandemie wenig untersucht (Denninger und Käpplinger 2021). Auch neben diesen Krisen bzw. Auslösern auf der Ebene der Gesellschaft existieren auf der individuellen Ebene kritische Ereignisse wie Krankheiten (Nittel und Seltrecht 2013), Erwerbslosigkeit (Matuschek und Stanik 2021) oder Veränderungen in sozialen Beziehungen durch Tod, Scheidung oder Wohnortswechsel (Sautermeister 2021), die für Betroffene Veränderungsnotwendigkeiten evozieren (Maier-Gutheil 2015, S. 10 f.). Rosenberg und Hof (2021) stellen bereits heraus, dass solche Ereignisse nicht per se mit negativen Ereignissen gleichzusetzen sind, sondern vielmehr in ihren „subjektiven Bewertungen“ (ebd., S. 12) als signifikante Veränderungen bzw. Infragestellungen von Handlungsroutinen auftreten und so als Lernanlässe fungieren können. Daraufhin werden womöglich transformatorische Lernprozesse initiiert (Koller 2018), die in der Entscheidung für ein ehrenamtliches Engagement als potenzieller Lernraum münden, um sich Bewältigungsstrategien für kritische Ereignisse anzueignen. Die Rolle freiwilligen Engagements hierbei ist in der Erwachsenenbildungsforschung bislang weitgehend unbeachtet. Die vorliegende Untersuchung soll einen Beitrag dazu leisten, diese Forschungslücke theoretisch und empirisch fundiert zu schließen. Eine solche Betrachtung freiwilligen Engagements als eine exemplarische Handlungsform der Bewältigung kann dazu beitragen, anhand der biografischen Einbettung bislang weitgehend isoliert untersuchter Motive (Clary et al. 1998) ein tieferes Verständnis für die Grundlagen freiwilligen Engagements zu erhalten sowie diesem eine neue Relevanz im Hinblick auf eine transformatorische Erwachsenenbildung zukommen zu lassen.

Zu diesem Zweck betrachtet die Studie die individuellen Einstellungen (Chell 2004) und subjektiven Wahrnehmungen kritischer Lebensereignisse (ebd.; Trautwein und Bosse 2017). Als Folgen dieser Ereignisse werden zunächst eine veränderte Lebenssituation der Betroffenen sowie im weiteren Verlauf die potenzielle Entscheidung für ein freiwilliges Engagement als eine exemplarische Handlungsform der Bewältigung angenommen. Konkret wird untersucht, inwiefern kritische Ereignisse für die Befragten in einem Zusammenhang mit ihrer Aufnahme eines Ehrenamtes stehen und inwiefern sie mit Veränderungen in der Lebenssituation einhergehen. Außerdem wird analysiert, inwiefern die Aufnahme eines freiwilligen Engagements den Befragten dazu dient, das von ihnen erlebte kritische Ereignis bewältigen zu lernen.

**Figure 1:**
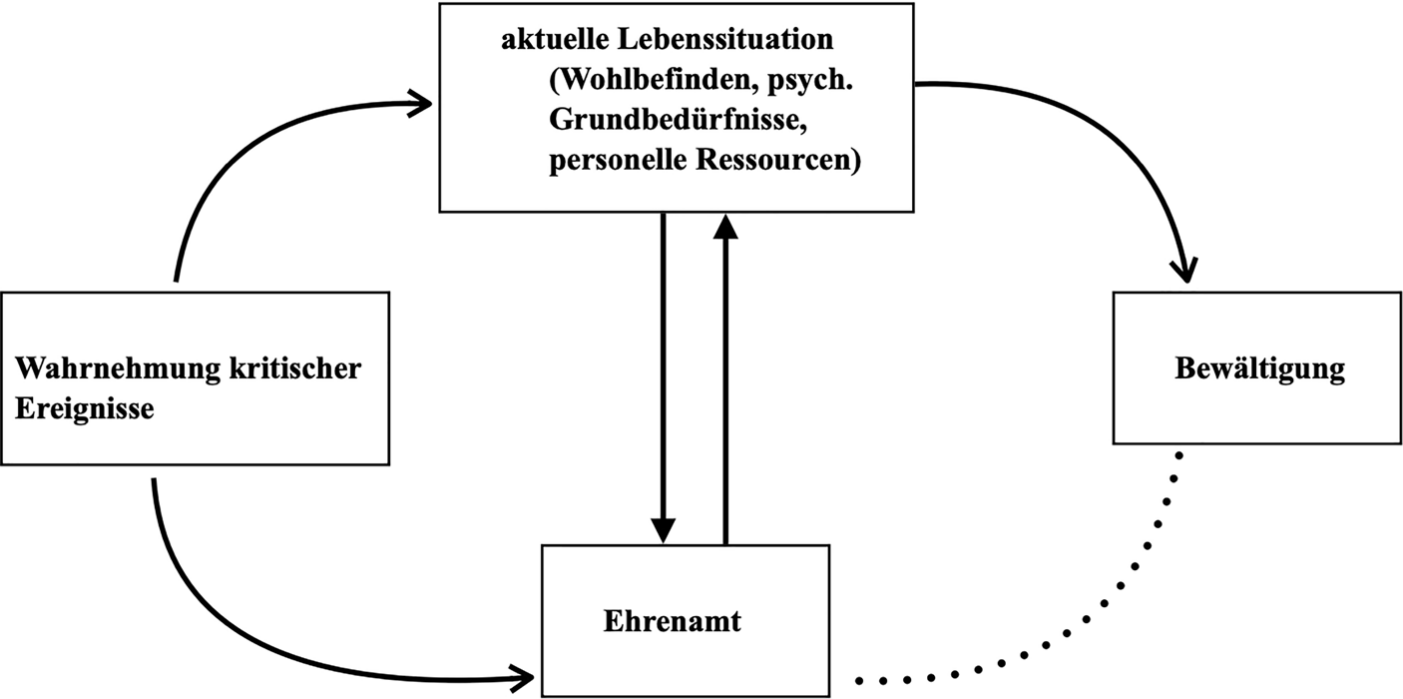

Zu den relevanten Faktoren der aktuellen Lebenssituation, welche unter dem Einfluss kritischer Ereignisse betrachtet werden, zählen die Befriedigung der psychologischen Grundbedürfnisse nach Kompetenz, Autonomie und Zugehörigkeit (Ryan und Deci 2017), das subjektive Wohlbefinden sowie personelle Ressourcen, von denen ausgehend motivationale Aspekte der Aufnahme eines freiwilligen Engagements wie z. B. Altruismus oder selbstbezogene Motive betrachtet werden (Emrich und Pierdzioch 2014; Clary et al. 1998; Abb. 1).

Die Untersuchung zielt darauf ab, anhand einer empirisch-fundierten Typenbildung die verschiedenen Aspekte der aktuellen Lebenssituation Erwachsener als Konsequenz wahrgenommener kritischer Lebensereignisse mit den davon abhängigen motivationalen Aspekten ihrer Aufnahme eines ehrenamtlichen Engagements ins Verhältnis zu setzen. Dazu wird eine explorativ-qualitative Analyse von Interviews mit Menschen vorgenommen, welche sich für die Aufnahme eines freiwilligen Engagements entschieden haben und unmittelbar vor dem Beginn ihrer ehrenamtlichen Tätigkeit stehen. Ausgehend von der Darstellung der relevanten theoretischen Konzepte werden im Weiteren die Forschungsfragen und das methodische Vorgehen erläutert. Im darauffolgenden Abschnitt werden die Ergebnisse der kategorienbasierten Auswertung sowie der Typenbildung dargestellt, um die verschiedenen Muster der Bewältigung kritischer Ereignisse durch ehrenamtliches Engagement aufzuzeigen. Im letzten Abschnitt werden diese Ergebnisse im Hinblick auf aktuelle theoretische sowie empirische Bezüge diskutiert.

## Theoretischer Hintergrund

Für die Untersuchung des komplexen Verhältnisses der subjektiven Wahrnehmung kritischer Lebensereignisse und deren Bewältigung bei Erwachsenen greift die vorliegende Arbeit zunächst Perspektiven transformativer Lern- und Bildungstheorien auf, mit denen einerseits angesichts kritischer Ereignisse die Bedingungen der Lebenspraxis und Lebenssituation als Ausgangspunkt transformativen Lernens betrachtet werden und andererseits die Bewältigung im Sinne einer transformativen Lernform untersucht werden kann. Mit solchen Lern- und Bildungsprozessen im Lebenslauf (Fuhr 2018) verortet sich der Beitrag in einer langjährigen biografischen Perspektive der Erwachsenenbildung (Nittel 2018; Kade und Nolda 2015), um gezielt diejenigen nicht institutionell konzeptualisierten Lernprozesse in den Blick zu nehmen, welche in bisherigen Konzepten des Lebenslangen Lernens nicht abgedeckt werden. Damit wird an biografisch informierte Einsichten der Erwachsenenbildung angeknüpft, Lernnotwendigkeiten für Individuen im Zuge biografischer Krisen anzunehmen und „quasi“ mitlaufende biografische Lernprozesse explizieren zu wollen (Alheit und Dausien 2006, S. 433). In Bezug auf solch krisenhafte Lernanlässe vollziehen sich während des Lernprozesses im Individuum häufig grundlegende Veränderung des bisherigen Denkens im Gegensatz zu einem ständigen *Dazu-Lernen* (Fuhr 2018). Günther Bucks Konzept der „negativen Erfahrung“ (2019) versucht, im Rahmen seiner epagogischen Erfahrungs- und Lerntheorie irritierende Ausnahmesituationen im Sinne eines „Um-Lernens“ zu erfassen, wobei die Erfahrung die „Aneignung von Neuem, noch Unbekanntem aufgrund schon Bekanntem“ sei (ebd., S. 8). So tritt das kritische Ereignis unabhängig von seiner subjektiv wahrgenommenen positiven bzw. negativen Beschaffenheit (Trautwein und Bosse 2017, S. 374) laut Koller (2022) durch eine außergewöhnliche Signifikanz hervor. Dabei werden „Grundfiguren des Welt- und Selbstverhältnisses“ (ebd., S. 20) sowie „Routinen, Gewohnheiten und Erfahrungswerte“ (Oevermann 1991, S. 297) durch Fremdheitserfahrungen herausgefordert und gehen häufig zunächst mit Gefühlen von Angst, Wut, Schuld oder Scham einher (Mezirow 2000, S. 22). Besonders fruchtbar für die Untersuchung von transformativen Lernprozessen im Zuge kritischer Ereignisse erscheinen Ulrich Oevermanns Überlegungen (1991, S. 269 ff.), in welchen das kritische Ereignis ein strukturelles Element der Entstehung des Neuen darstellt. Demnach besteht die Bewältigung kritischer Lebensereignisse im Sinne einer Transformation zunächst aus der intuitiven Produktion „innerer Bilder“ hinsichtlich dessen, was zur Krisenbewältigung fehlt (ebd., S. 316). Anschließend vollzieht sich eine Übersetzung solcher „inneren Bilder“ in realitätsgerechte Krisenlösungen (Oevermann 1991, S. 314 ff.).

Nach Oevermann (1991, S. 304) ist die Entstehung des Neuen immer ein motivierter Vorgang. Davon ausgehend erscheint eine motivationstheoretische Betrachtung der Aufnahme eines freiwilligen Engagements als lohnenswert, welche zugleich die subjektiv wahrgenommenen Auswirkungen kritischer Ereignisse auf die Lebenssituation der Betroffenen als motivationale Grundlage ihres Handelns berücksichtigt. Die Selbstbestimmungstheorie – oder im Einzelnen die Basic Psychological Needs Theory (BPNT) – von Ryan und Deci (2017) ermöglicht es, ausgehend von spezifischen Zuständen der Befriedigung psychologischer Grundbedürfnisse nach Kompetenz, Autonomie und Zugehörigkeit Rückschlüsse auf das subjektive Wohlbefinden von Personen sowie die intrinsischen und extrinsischen motivationalen Grundlagen ihres Handelns zu ziehen. Ergänzend dazu fokussiert die Unterscheidung zwischen expliziten und impliziten Motiven (McClelland et al. 1989) personelle Faktoren, die einerseits als unbewusste affektive Prägungen (implizite Motive) vorliegen können und sich andererseits orientierend bzw. energetisierend auf das Verhalten auswirken (Heckhausen und Heckhausen 2018, S. 4 f.; Brunstein 2018, S. 369 ff.). Mit seiner Betrachtung der Aktivierungsdimension von Emotionen bekräftigt Pekrun (2018) die affektiven Grundlagen kognitiver Prozesse wie etwa Motivation, wobei sowohl positive Emotionen wie Freude als auch negative Emotionen wie Angst aktivierend wirken und zum Handeln motivieren können. Dahingegen orientieren sich explizite Motive an normativen Erwartungen, Wertungen und Einstellungen. Sie sind in der Regel bewusst zugänglich und wirken sich regulierend auf das Verhalten aus (Brunstein 2018, S. 369 ff.; McClelland et al. 1989).

Treten motivationale Orientierungen in verbalisierter Form in Erscheinung, geschieht dies häufig im Rahmen der Schilderung von Handlungserfahrungen, welche in der Vergangenheit gemacht worden sind. Dabei werden von Betroffenen retrospektive Deutungen und Umdeutungen von Interaktionsgeschichten vollzogen, welche den gesamten Vorstellungsraum der Lebensführung überformen (Matthes und Schütze 1980, S. 38 f.). Motiv-Begründungen entfalten sich hierbei einerseits in Form verstehbarer Anfänge von Ursachen einer Handlungssequenz oder eines Handlungsentwurfes („weil-Motive“) und andererseits in Form von zukunftsorientierten Zielerfüllungen oder Realisierungszwecken („um-zu-Motive“; Schütz 1972, S. 13 ff.).

## Fragestellung und Methode

Die qualitativ-explorative Studie zielt darauf ab, das Verhältnis kritischer Lebensereignisse zur aktuellen Lebenssituation unter Erwachsenen aufzuarbeiten. Davon ausgehend will sie subjektive Implikationen für die motivationalen Aspekte der Aufnahme eines freiwilligen Engagements identifizieren. Dazu widmete sie sich der folgenden Fragestellung:

1. *Inwieweit kann ehrenamtliches Engagement als ein Muster der Bewältigung kritischer Lebensereignisse angesehen werden?*

Für eine solche Betrachtung subjektiven Sinns und sozialer Rekonstruktion von Wirklichkeit in Bezug auf das Verhältnis von kritischen Lebensereignissen und der Aufnahme freiwilligen Engagements wird die Methode des episodischen Interviews gewählt (Flick 2010, 2011). Dabei werden zur Zeit des zweiten und dritten Lockdowns, zwischen Oktober 2020 und November 2021, insgesamt neun episodische Interviews mit Menschen geführt, die sich kürzlich für eine ehrenamtliche Tätigkeit entschieden haben. Das heißt, die Teilnehmenden werden danach ausgewählt, ob sie sich am Beginn ihres Ehrenamtes befinden, wobei nicht danach gefragt wird, ob kritische Lebensereignisse in der jüngsten Vergangenheit aufgetreten sind. Die Rekrutierung der Ehrenamtlichen erfolgt über Multiplikatoren^1^, wobei Ehrenamtliche mit unterschiedlichen soziodemografischen Merkmalen (Alter, Geschlecht, etc.) angesprochen werden. Um narratives Wissen zu erheben, werden zunächst offene Fragen gestellt bzw. situationsbezogene Erzählanstöße gegeben (Flick 2011). Diese beziehen sich auf kritische Ereignisse im Vorfeld des Engagements und deren Einfluss auf die aktuelle Lebenssituation (Wohlbefinden und Befriedigung psychologischer Grundbedürfnisse), auf den Entscheidungsprozess in Bezug auf die Aufnahme des Engagements sowie auf den ersten Kontakt zur Einrichtung des künftigen Engagements. Im weiteren Verlauf des Interviews wird semantisches Wissen zu den Phänomenen „kritische Lebensereignisse“, „Bewältigung“ und „Ehrenamt“ erfasst. Ein solcher Abgleich von narrativem mit semantischem Wissen bietet einen wichtigen Anhaltspunkt für die Interpretation von impliziten Strukturen, wie etwa impliziter Motive (McClelland et al. 1989). Zur computergestützten Durchführung der qualitativen Inhaltsanalyse (Kuckartz 2018) wird die Software MAXQDA verwendet, wobei sich der induktiv-deduktive, kategorienbasierte Analyseprozess aus einem inhaltlich-strukturierenden Teil (Kuckartz 2018), gefolgt von einer typenbildenden Inhaltsanalyse zusammensetzt (Kuckartz 2018; Kelle und Kluge 2010). Auch wenn aus Ressourcengründen kein weiterer Codierer in die Auswertung miteinbezogen werden kann, wird das gesamte Material nach 3‑monatiger Pause erneut codiert. Dabei werden Übereinstimmungen bzw. Differenzen zwischen beiden Codierdurchgängen identifiziert und reflektiert, um die Zuverlässigkeit der Inhaltsanalyse zu erhöhen und der Reliabilität des Forschungsdesigns Rechnung zu tragen (Kuckartz 2018, S. 202). Alle Kategorien des Kategoriensystems werden vorab definiert und mit Ankerbeispielen versehen. Zudem werden Fallzusammenfassungen erstellt und im Rahmen der initiierenden Textarbeit wird mit Memos gearbeitet.

Als erster Schritt der Typenbildung wird anhand der Forschungsfrage ein Merkmalsraum aus konstituierenden Merkmalen gebildet (Kuckartz 2018). Das für die Typenbildung herangezogene Material wird anhand entsprechender thematischer Haupt- und Subkategorien ausgewählt, mit denen der gesamte Datensatz bereits codiert wurde. Anschließend werden die Ausprägungen der zwei Dimensionen des Merkmalsraumes im Sinne einer Vierfeldertafel (Kuckartz 2018, S. 149) miteinander gekreuzt und die einzelnen Fälle entsprechend der zutreffenden Kategorien darauf verortet, woraus schließlich die empirisch begründeten Typen abgeleitet werden. Anschließend werden die gebildeten Typen entsprechend der relevanten Ausprägungen im Detail beschrieben (Kuckartz 2018, S. 157). Um die Merkmale der Typen in ihrer Spezifik trotz des hohen Interpretationsbedarfs abbilden zu können, wird schließlich auf soziodemografische Informationen eingegangen und es werden Überlegungen zu Zusammenhängen zwischen den Typen und sekundären Informationen angestellt (Kuckartz 2018, S. 158 f.). Zudem wird in einem zusätzlichen Kurzfragebogen nach der „aktuellen Lebenssituation“ gefragt. Dazu wird die gegenwärtige Befriedigung der psychologischen Grundbedürfnisse auf einer Ratingskala erfasst, um anhand von Mittelwerten ebenfalls Rückschlüsse auf das Wohlbefinden der einzelnen Typen zu gewinnen (Ryan und Deci 2017, S. 11).

## Ergebnisse: Kritische Ereignisse, die aktuelle Lebenssituation und Engagement-Motive im Überblick

Tab. 1 soll einen Überblick über kritische Lebensereignisse bzw. deren Verkettung in den einzelnen Fällen des Samples geben. Ausgehend davon werden die aktuelle Lebenssituation der Betroffenen und davon abhängige Motive für die Aufnahme eines Engagements aufgezeigt. Die Darstellung dient in der sich anschließenden Typenbildung dazu, die verschiedenen Muster der Bewältigung kritischer Lebensereignisse durch freiwilliges Engagement mit konkreten Ereignissen der einzelnen Fälle sowie spezifischen Faktoren der aktuellen Lebenssituation zu unterlegen.

**Table 1:** 

Fälle	Abfolge der Ereignisse	Engagement-Form
B1	*Pflege der Mutter* → Umzug der Tochter ins Ausland → Erfahrungen in Pflegeeinrichtungen → Tod der Mutter → Umzug in neue Wohnung → *Ankunft in der neuen Wohnung*	Hospizarbeit
B2	Selbstständigkeit → *Corona* → *Aufgabe der Selbstständigkeit* → *Indien-Reise* → *Hilfsbereitschaft von Fremden* → *Reflexion des eigenen Egos*	Hospizarbeit
B3	Corona/Lockdown/Sturz in der U‑Bahn → körperliche Einschränkungen → Alter/Ende professioneller Arbeit → *Reflexion der eigenen Bedeutsamkeit*	Hospizarbeit
B4	Tod eines Freundes → Umzug nach Berlin → Corona/Lockdown	Hospizarbeit
B5	Affäre des Ehepartners → Trennung/Scheidung → *Therapie* → *Reflexion der früheren Lebenssituation (Beziehung, privat, Beruf)*	Hospizarbeit
B6	*Umzug nach Berlin* → Unzufriedenheit im neuen Job → Corona/Lockdown → *Personalgespräch* → *interner Jobwechsel*	Kinder- und Jugendhilfe
B7	Umzug nach Berlin → Corona/Lockdown → Abschiedsfeier/*letztes Ehrenamt* → Unzufriedenheit im Job	Nachbarschaftsarbeit
B8	Trennung von Partnerin → Tod des Vaters → Erbstreit → Umzug in neue Wohnung → Streit mit ehem. Vermieter → Konflikte mit Schwiegertochter → Streit zwischen enger Freundin und deren Tochter	Nachbarschaftsarbeit
B9	Sinnkrise → *Vipassanakurs* → *Corona-Lockdown* → *Ehrenamt bei Vipassana e.* *V.*	Kinder- und Jugendhilfe

Negativ-aktivierende Ereignisse, *positiv-aktivierende Ereignisse*

### Empirisch begründete Motiv-Typen freiwilligen Engagements

Zur Bildung der im Folgenden präsentierten Motiv-Typen freiwilligen Engagements werden die Kategorien „Bewertung der aktuellen Lebenssituation“ und „Motive“ als konstituierende Merkmale herangezogen (Tab. 2).

**Table 2:** 

	Bewertung der aktuellen Lebenssituation	
Motive	Positive Bewertung der Lebenssituation	Negative Bewertung der Lebenssituation
Altruismus	B2; B6; B9 *Typ 2*	B1; B5 *Typ 1*
Egoismus	–	B3; B4; B7; B8 *Typ 3*

### Typ 1 – Selbstsorge durch Fürsorge (*N* = 2)

Typ 1 repräsentiert weibliche Personen höheren Alters (∅ 59 Jahre alt) mit einer geringen wöchentlichen Arbeitsauslastung (∅ 26 h pro Woche), die sich aus tendenziell negativen Lebensumständen für die Aufnahme eines freiwilligen Engagements im Bereich der Sterbebegleitung entschieden haben. Dabei legen sie im Rahmen expliziter Selbstauskunft eine altruistische Orientierung in Bezug auf die eigenen Motive zum künftigen Engagement an den Tag, wie zum Beispiel „etwas leisten für die Allgemeinheit“ (B5, Z. 151), „eine Person, welche auch immer, ein Stück glücklicher machen“ (B1, Z. 375–376). Neben den explizit altruistischen Motiven zeigt Typ 1 implizit ein *ergänzendes Bewältigungsstreben* kritischer Lebensereignisse im Rahmen eines freiwilligen Engagements, welches sich aus den Handlungsursachen („weil-Motive“, Schütz 1972, S. 13) und den zukunftsorientierten Zielerfüllungen („um-zu-Motive“, ebd.) der Aufnahme eines freiwilligen Engagements im Rahmen retrospektiver Erzählungen zu kritischen Ereignissen und deren Auswirkungen auf die Lebenssituation zusammensetzt. Die „weil-Motive“ basieren auf Veränderungen in der Lebenssituation, dessen Faktoren sich einerseits durch ein Mehr an personellen Ressourcen wie einem Zugewinn an Freizeit auszeichnen. Andererseits zeigen Personen des ersten Typus eine Verminderung in der Befriedigung psychologischer Grundbedürfnisse nach Kompetenz und Zugehörigkeit, was mit einer Verringerung des subjektiven Wohlbefindens einhergeht. Diese Schilderungen zu den spezifischen Zuständen der aktuellen Lebenssituation verweisen gleichzeitig auf innere Bilder bzw. intuitive Vorstellungen gegenüber dem, was zur Bewältigung eines oder mehrerer kritischer Ereignisse ergriffen werden muss (Oevermann 1991). Die Lebensumstände von Typ 1 stehen unter dem Einfluss negativ-kritischer Ereignisse circa anderthalb bis zwei Jahre vor der Aufnahme des Engagements, welche als „Schicksalsschläge“ erlebt werden, wie z. B. der Tod eines Familienangehörigen (s. B1, Tab. 1) oder partnerschaftliche Schwierigkeiten (s. B5, Tab. 1), und mit negativ-aktivierenden Emotionen wie Ärger oder Trauer verbunden sind (Pekrun 2018).

„Die ist Anfang Dezember gestorben. (..) Und Ende Januar haben wir sie beerdigt. Das ging nicht vorher, weil auch wieder meine Tochter nicht kommen konnte, mit den Flügen. Und, das war nicht so einfach. Und da war es tatsächlich noch so. Da war jeden Tag ein Loch.“ (B1, Z. 227–230)

Aus diesen Lebensumständen und emotionalen Ausgangslagen ergreift Typ 1 bereits erste Bewältigungsmaßnahmen im Vorfeld des Engagements, wie etwa eine Therapie (s. B5, Tab. 1) oder den Umzug in eine neue Wohnung (s. B1, Tab. 1), wodurch bestehende Denkmuster und Routinen unterbrochen und Ansätze neuer Lebenspraktiken reflektiert werden.

„… durch die psychologische Begleitung usw., da ist es ja sowie nochmal, dass man viel mehr über sich selber nachdenkt. (..) *Insofern habe ich natürlich mich schon viel in den letzten anderthalb Jahren jetzt mit mir beschäftigt* … ist ja so ein Punkt, wo man so nochmal neu nachdenkt: „Wie sollte es jetzt kommen? Was könnte sich ändern?““ (B5, Z. 342–347)

Mithilfe dieser Maßnahmen nehmen die Personen einen positiven Einfluss auf die zunächst signifikant negativen Zustände der aktuellen Lebenssituation. An dieser Stelle fügen sich die „um-zu-Motive“ der Aufnahme eines freiwilligen Engagements an, die aus einem Abgleich von semantischem und narrativem Wissen zu kritischen Ereignissen mit Wissen zu freiwilligem Engagement evoziert werden. Das heißt, aus den Erfahrungen zu kritischen Ereignissen und ihren Auswirkungen auf die Lebenssituation leitet Typ 1 in Anbetracht antizipierter Anforderungen und Inhalte des künftigen Engagements Zielerfüllungen der Aufnahme im Sinne einer ergänzenden Bewältigung ab, wobei nach wie vor verringertes Wohlbefinden gefördert und ein ausgeglichenes Kompetenz- bzw. Zugehörigkeits-Erleben gestärkt werden soll (Benedetti 2015; Düx et al. 2009, S. 43 f.). Die Entscheidung für ein freiwilliges Engagement bei Typ 1 – *Selbstsorge durch Fürsorge* – im Sinne eines lohnenswerten Bewältigungsformates zeigt sich zudem insbesondere vor dem Hintergrund der biografischen Einordnung des semantischen und narrativen Wissens zu freiwilligem Engagement. Hierbei werden biografische Erfahrungen, wie beispielsweise die „Pflege der eigenen Mutter“ (s. B1, Tab. 1) oder eine Dokumentation über „ehrenamtliche Sterbebegleitung mit Hunden“, sichtbar, welche der Entscheidung für ein Engagement in der Sterbebegleitung eine hohe Übereinstimmung mit dem, was für die Bewältigung kritischer Lebensereignisse ergriffen werden muss, zugestehen.

„Aber, habe dort, gerade weil ich um die Mittagszeit da war, ganz viel Kontakt zu den anderen Mitbewohnern gehabt … Und, teilweise haben die mir ihre halben Lebensgeschichten erzählt und ich habe dann immer so bei mir gedacht: „Es ist so wichtig, dass die jemanden haben, der auch mal (.) einfach nur zuhört.“ … Und haben die immer alle gesagt: „Boah, (Name B1). Wenn Sie mal nicht mehr kommen. Sie werden uns ja alle fehlen.““ (B1, Z. 201–218)

### Typ 2 – Fürsorge aus Überzeugung (*N* = 3)

Bei Typ 2 handelt es sich um weibliche Personen jüngeren Alters (∅ 30 Jahre alt) mit einer hohen wöchentlichen Arbeitsauslastung (∅ 37,3 h pro Woche), die sich aus einer positiven Lebenssituation für die Aufnahme eines freiwilligen Engagements im Bereich der Sterbebegleitung, bzw. Kinder- und Jugendhilfe entschieden haben und dabei explizit altruistische Motive schildern: „*Ich glaube, die Funktion ist (..) anderen zu helfen und zwar ohne selber was dafür in* irgendeiner* Form zu erwarten.“* (B9, Z. 1188–1189). Ergänzend dazu zeigt sich bei der Aufnahme eines freiwilligen Engagements implizit ein *vergewisserndes Handlungsmuster*. Die „weil-Motive“ dieses Musters gehen auf eine signifikant positive Lebenssituation mit hohem subjektivem Wohlbefinden, einer ausgeglichenen Befriedigung psychologischer Grundbedürfnisse, einem Zugewinn an Freizeit sowie der Reflexion sinnstiftender Themen, wie etwa der Reflexion des eigenen Egos zurück, denen sich im Zuge des künftigen Engagements handelnd vergewissert werden soll.

„Und (..) ich finde das auch wirklich eine schöne Herausforderung für mich, dass es tatsächlich in dieser Zeit nur um den anderen Menschen geht. Es geht nicht um mich, es geht nicht um mein Ego …“ (B2, Pos. 186–188)

Die positiven Lebensumstände von Typ 2 stehen unter dem Einfluss eines, bzw. mehrerer als signifikant positiv erlebter Ereignisse, wie beispielsweise „Hilfsbereitschaft von Fremden“ (s. B2, Tab. 1), die mit positiv-aktivierenden Emotionen wie Freude, Hoffnung oder Stolz einhergehen (Pekrun 2018). Dabei fällt auf, dass die Corona-Pandemie und deren Auswirkungen im Unterschied zu Typ 1 und Typ 3 hier im Sinne einer „Wildcard“ als Türöffner für weitere positive Ereignisse erlebt wurde. Dabei wurde zunächst eine negative Ausgangssituation, wie zum Beispiel eine persönliche Sinnkrise oder ein belastendes Arbeitsverhältnis, unterbrochen (s. B9/B2, Tab. 1), womit gleichzeitig Spielräume für innovative Prozesse (Oevermann 1991, S. 116) in Form von positiv-kritischen Ereignissen eröffnet wurden, wie beispielsweise eine „Indienreise“, „Hilfsbereitschaft von Fremden“ oder der „Beginn einer neuen Lebenspraxis“ (s. B9, Tab. 1).

„Es war gerade ganz frisch Corona. *Oh ja, da ging es mir super (lacht).* Ne, ganz frisch Corona war, für mich war es gut. Es hat mir so einen cut reingeschnitten in was, was sowieso gut war, dass das jemand gecuttet hat. *Joa, das kommt hin*. (6)“ (B9, Z. 87–91)

Die „um-zu-Motive“ des *vergewissernden Handlungsmusters* basieren auf dem semantischen und narrativen Wissen von Typ 2 zu freiwilligem Engagement. So verweisen die antizipierten Anforderungen und Inhalte des künftigen Engagements auf die Möglichkeit, sich einerseits über die Weitergabe der eigenen Fähigkeiten und Talente der eigenen Ressourcen gewahr zu werden und andererseits im Sinne eines altruistischen Gefühls der Verantwortung für Mitmenschen oder die Umwelt gesellschaftliche Herausforderungen zu bearbeiten (Düx et al. 2009, S. 182 ff.). Somit handelt Typ 2 – *Fürsorge aus Überzeugung* – bezüglich der Aufnahme des Engagements aus einer positiven Lebenssituation heraus, welche von der Überzeugung begleitet ist, die eigenen Ressourcen mit anderen teilen zu wollen oder gar zu müssen.

„Egal, was du tust, es ist immer mehr Arbeit und immer mehr Arbeit und immer mehr Arbeit *und du bist nie fertig, nie*. Weil es immer noch mehr zu tun gibt … und dann geht es einem gut und anderen geht es auch gut und das ist, glaube ich, der Sinn des Lebens, wenn man so will. Also, vielleicht noch ein bisschen mehr als das, aber ich glaube, das ist die grund-solide Basis für alles andere (lacht).“ (B9, Z. 1218–1228)

### Typ 3 – Fürsorge als Selbstsorge (*N* = 4)

Typ 3 umfasst weibliche und männliche Personen, die sich erst kürzlich örtlich niedergelassen haben (∅ 1 Jahr) und sich aus einer signifikant negativen Lebenssituation für die Aufnahme eines freiwilligen Engagements im Bereich der Sterbebegleitung bzw. Nachbarschaftsarbeit entschieden haben. Bezüglich des künftigen Engagements äußert Typ 3 explizit selbstbezogene Motive wie das „Knüpfen sozialer Kontakte“ (B8, Z. 348) oder „Wertschätzung“ (B7, Z. 166). Ergänzend zu diesen Motiven zeigt sich bei Typ 3 ein explizites und *akutes Bewältigungsmuster* kritischer Ereignisse bei der Aufnahme eines freiwilligen Engagements. Die „weil-Motive“ dieses Bewältigungsmusters basieren auf negativen Zuständen der aktuellen Lebenssituation mit signifikant verringertem Wohlbefinden, einem niedrigen Kompetenz‑, Autonomie- und Zugehörigkeitserleben sowie teilweise körperlichen Einschränkungen. Hierbei stehen die Lebensumstände unter dem Einfluss von als signifikant negativ erlebten kritischen Ereignissen, wie der „Corona-Pandemie“, einem „Unfall“ oder „zwischenmenschlichen Konflikten“ (s. B8/B7/B4/B3, Tab. 1), welche negativ-aktivierende Emotionen wie Trauer, Ärger und Angst evozieren (Pekrun 2018).

„Also, am vierten März (lacht) habe ich einen schweren Sturz erlitten … das korrespondierte so mit diesem Corona, mit der Corona. Deshalb muss ich das *erwähnen*. Weil dadurch, weil ich *körperlich* ausgeknockt war und hätte auch alles, was ich an Kurse und so, absagen müssen, was dann durch Corona auch passiert ist. Das ist, deshalb muss ich das erwähnen, das ist so deckungsgleich. Ich war sozusagen aus meinem alltäglichen Aktivismus (.) rausgeschuppst.“ (B3, Z. 92–100)

Die Verkettung der negativ-kritischen Ereignisse beginnt circa anderthalb Jahre vor Beginn des Engagements und erstreckt sich bis zu dessen Aufnahme, wodurch dieser Moment durch die negativ-aktivierenden Emotionen sowie Zustände der negativen Lebenssituation eingefärbt ist. Die „um-zu-Motive“ des akuten Bewältigungsmusters basieren auf dem semantischen und narrativen Wissen von Typ 3 zu freiwilligem Engagement. Dabei fällt auf, dass dieses Wissen entweder durch langjährige, positive Erfahrungen in früheren Ehrenämtern oder durch langjährige Erfahrungen in dem künftigen Engagement ähnlichen Tätigkeitsfeldern angereichert ist.

„Ja, ich kann ja wieder über die, mein Engagement von vorher dann reden. Bei uns gab es immer so Sommerlager. Das waren zehn bis fünfzehn Tage, wo man sich um die Kinder kümmerte. Und das war natürlich auch mal eine ganze Bewältigung … am Ende haben wir das dann auch zusammen geschafft dann … *bei mir ist Engagement auf jeden Fall Ehrenamt (…) so ein falscher Egoismus*. Man macht es zwar für die anderen, aber man macht es vor allem aber auch, weil man sich selber dann besser fühlen kann.“ (B7, Z. 541–550)

Daher liegen die individuellen Erträge sowie das Potenzial eines freiwilligen Engagements als Bewältigungsmodus kritischer Ereignisse einerseits reflektiert und explizit vor, wovon Bewältigungsbestrebungen im Rahmen des künftigen Engagements über das Knüpfen sozialer Kontakte, die Wertschätzung der eigenen Fähigkeiten und Talente sowie persönliches Wachstum abgeleitet werden (Benedetti 2015; Düx et al. 2009, S. 141 ff.). So deutet bei Typ 3 die Aufnahme eines freiwilligen Engagements in Anbetracht dieser biografischen Erfahrungen in ihrer Funktion auf ein realitätsgerechtes Mittel der Krisenlösung hin (Oevermann 1991).

## Zusammenfassung, Diskussion und Ausblick

In diesem Abschnitt werden zunächst die Untersuchungsergebnisse in Anbetracht des Anliegens der Studie zusammengefasst. Weiterhin werden die vorliegenden Ergebnisse bezüglich aktueller Befunde reflektiert, um an den Diskurs zu transformatorischen Bildungsprozessen anzuschließen und den Beitrag für die Erwachsenenbildungsforschung aufzuzeigen.

Das Ziel der Untersuchung war es, empirisch begründete Motiv-Typen freiwilligen Engagements in Abhängigkeit zur aktuellen Lebenssituation von erwachsenen Ehrenamtlichen zu identifizieren. Zudem sollte untersucht werden, inwiefern kritische Lebensereignisse bei Ehrenamtlichen vorliegen und welcher Zusammenhang zwischen diesen Ereignissen, der aktuellen Lebenssituation und der Entscheidung für ein Ehrenamt besteht. Damit sollte der Frage nachgegangen werden, inwieweit ehrenamtliches Engagement als eine Form der Bewältigung kritischer Lebensereignisse angesehen werden kann. Auf der Basis einer typenbildenden qualitativen Inhaltsanalyse wurden drei Typen gefunden, die sich darin unterscheiden, inwieweit ein Ehrenamt als Teil von Bewältigungsprozessen betrachtet wird: Typ 1 *– Selbstsorge durch Fürsorge*, Typ 2 – *Fürsorge aus Überzeugung *und Typ 3 *– Fürsorge als Selbstsorge*. Die Label der identifizierten Typen sind anschlussfähig an Foucault’s Überlegungen zur „Sorge um sich selbst“, in denen verschiedene Logiken und Praktiken darauf abzielen, die Beziehung zwischen Subjekt und Wahrheit aufzuklären (Foucault 2009, S. 16). Dieses Konzept wird hier mit fürsorgerischen Praktiken und Logiken innerhalb prosozialer Engagement-Formen wie Sterbebegleitung, Kinder- und Jugendhilfe oder Nachbarschaftsarbeit in Beziehung gesetzt. Die vorliegenden Motiv-Typen verweisen auf Tätigkeiten der Befragten, bei denen sie explizite Werte und Überzeugungen mit einer implizit heilenden bzw. teilweise therapeutischen Wirkung für sich selbst vereinen.

Mit Blick auf transformatorische Bildungstheorien zeigen die Ergebnisse, dass sich Menschen im Vorfeld ihres Engagements unter dem Einfluss kritischer Lebensereignisse befinden können, die bestehende Welt- und Selbstverhältnisse sowie Routinen in Frage stellen und diese gar unterbrechen (Koller 2018; Oevermann 1991). Hinzu kommt, dass sich transformative Lernprozesse laut Mezirow (2000, S. 22) häufig dem Vorhandensein von Emotionen wie Wut oder Angst anschließen – die vorliegenden Motivtypen zeigen sowohl positive (Freude, Stolz) als auch negative Emotionen (Angst, Wut). Pekrun (2018) bekräftigt, dass sowohl negative als auch positive Emotionen von besonderer Bedeutung für Motivation, Aufmerksamkeit sowie Handlungsentscheidungen sind und demnach als Ausgangspunkt für transformative Lernprozesse betrachtet werden könnten. Obwohl die Erfahrung eines kritischen Lebensereignisses laut Oevermann einen zentralen Schritt in der Verlaufsstruktur von Transformationsprozessen ausmacht (Koller 2018, S. 119), ist dennoch nicht per se davon auszugehen, dass kritische Lebensereignisse in die Entscheidung für ein freiwilliges Engagement als Gegenstandsbereich transformativer Bildungsprozesse münden, um im Laufe des Engagements neue Figuren von Welt- und Selbstverhältnissen zu entwickeln (Koller 2022). Auch Depression, Gelähmtheit und Verwirrung im Sinne eines „Nichtlernens“ (Nittel 2013, S. 157 ff.) oder die Restabilisierung etablierter Welt- und Selbstbezüge (Koller 2022, S. 17) können mögliche Antworten auf kritische Lebensereignisse sein. Es stellt sich also die Frage, von welchen Bedingungen eine Initiierung transformativer Lernprozesse im Zuge freiwilligen Engagements abhängt. Es scheint, dass es vor dem Hintergrund biografischer Erfahrungen in früheren Ehrenämtern, beruflichen Tätigkeiten oder zwischenmenschlichen Beziehungen zu einem Abgleich zwischen bereits Erlebtem und gegenwärtig Fehlendem kommt. Dabei stehen „innere Bilder“ von dem, was zur Bewältigung ergriffen werden muss – als Produkt der aktuellen Lebenssituation – den antizipierten Anforderungen und Inhalten des künftigen Engagements gegenüber. In diesem Zusammenhang kann es laut Düx et al. (2009, S. 43 f.) zur Aufnahme eines Engagements kommen, „wenn in einer spezifischen Lebensphase Motiv, Anlass und Gelegenheit biografisch zusammenpassen.“ Das heißt, die Bezugnahme auf biografische Erfahrungen lässt Bedingungen sichtbar werden, durch die der Aufnahme eines freiwilligen Engagements eine bildungsbiografische Bedeutung zukommt (Keil 2013), weil sie als eine realitätsgerechte Krisenlösung erscheint (Oevermann 1991, S. 314 ff.).

Somit exploriert die Arbeit anhand der vorliegenden Motiv-Typen drei typische Konstellationen der Aufnahme eines freiwilligen Engagements, in denen sich die subjektive Wahrnehmung kritischer Ereignisse, deren Auswirkung auf die aktuelle Lebenssituation und die bisherige Lebensgeschichte unterschiedlich verorten. Anhand dieser Konstellationen lassen sich gleichfalls theoretisch wertvolle Lernziele im gemeinsamen Modus des „Umlernens“ identifizieren, in dem ein bereits vorhandener Korpus an Kompetenzen und Wissen auf einen anderen Gegenstands- oder Handlungsbereich, wie hier das freiwillige Engagement, übertragen wird (Nittel 2013, S. 145). Demnach lernt Typ 1, positive Handlungsoptionen im Zuge erlittener Verluste von „signifikanten Anderen“ (Familienangehörige, Ehepartnerinnen und -partner, ebd., S. 153) durch Tod bzw. Scheidung zu erkennen, diese für das eigene Wohlbefinden zu nutzen sowie sich selbst ohne die einstigen Angehörigen im eignen Lebenslauf zu positionieren. Es lässt sich zudem vermuten, dass Typ 1 im Verlauf des Lernens gezielt an Erfahrungen als pflegende Angehörige oder Ehepartnerin anknüpft, um diese im freiwilligen Engagement zu erweitern, zu reflektieren und Einzelnes ggf. zu überwinden. Typ 2 lernt, neue Handlungsoptionen und Ressourcen im Zuge positiv-erlebter Ereignisse, wie die COVID-19-Pandemie, hinsichtlich der eigenen Werte und Einstellungen umzusetzen. Hierbei lässt sich vermuten, dass sich das Lernen ausgehend von einer negativen Lebenssituation im weiteren Verlauf in einer positiven Verlaufskurve mit einer Verkettung mehrerer, als positiv erlebter Ereignisse (Nittel 2013) befindet, aus der die Entscheidung für ein freiwilliges Engagement geschieht. Typ 3 lernt, mit den Konsequenzen negativer Ereignisse umzugehen. Dabei deutet sich im Verlauf des Lernprozesses an, dass Erfahrungen positiver Erträge aus früheren Ehrenämtern oder beruflichen Kontexten aufgegriffen werden, um im freiwilligen Engagement negative Konsequenzen kritischer Ereignisse zu überwinden.

In Bezug auf weitere Studien implizieren die Ergebnisse eine lohnenswerte biografisch-vertiefende Untersuchung der Typologie, um die Motiv-Typen mit biografischen Verlaufskurven kritischer Ereignisse in Beziehung zu setzen und die Rolle freiwilligen Engagements i. S. einer Bewältigung für verschiedene Lebensläufe abzubilden. Für die gezielte Ansprache neuer Engagierter bedeuten die vorgelegten Typen eine besondere Sensibilität des hauptamtlichen Personals für deren individuelle Hintergründe, um Engagementformen zu vermitteln, die eine hohe Passgenauigkeit zwischen der Lebenssituation sowie den davon ausgehenden Motiven und den Anforderungen des Engagements sicherzustellen und dadurch bildungsbiografisch bedeutsame Erfahrungen zu ermöglichen. Erwachsenenpädagogisch bekräftigen die Ergebnisse die Notwendigkeit, freiwilliges Engagement als Erfahrungsraum biografischer und transformativer Bildungsprozesse zu begreifen, welcher neben altruistischen Motiven auch latente, „quasi-therapeutische Zielsetzungen“ (Alheit und Dausien 2006, S. 433) umfasst und durchaus Auswirkungen auf formelle Erwachsenenbildungsbereiche hat (Benedetti 2015, S. 67).

## Footnotes

1 Es wurden Kooperationen mit verschiedenen Non-Profit-Organisationen in Berlin eingegangen. Dort tätige Ehrenamtskoordinierende sorgen für die Ansprache neuer Ehrenamtlicher bezüglich der Interviewteilnahme und vermittelten die Kontaktdaten.

## References

[CR1] Alheit P, Dausien B, Krüger H-H, Marotzki W (2006). Biografieforschung in der Erwachsenenbildung. Handbuch erziehungswissenschaftliche Biografieforschung.

[CR2] Benedetti S (2015). Freiwilliges Engagement – ein bildungsbiografischer Erfahrungsraum. Zeitschrift für Weiterbildungsforschung.

[CR3] Brunstein JC, Heckhausen H, Heckhausen J (2018). Implicit and explicit motives. Motivation and action.

[CR4] Buck G (2019). Lernen und Erfahrung. Epagogik.

[CR5] Chell E, Cassell C, Symon G (2004). Critical incident technique. Essential guide to qualitative methods in organizational research.

[CR6] Clary EG, Snyder M, Ridge RD, Copeland J, Stukas AA, Haugen J, Miene P (1998). Understanding and assessing the motivations of volunteers: A functional approach. Journal of Personality and Social Psychology.

[CR7] Denninger A, Käpplinger B (2021). COVID-19 und Weiterbildung – Überblick zu Forschungsbefunden und Desideraten. Zeitschrift für Weiterbildungsforschung.

[CR8] Düx W, Prein G, Sass E, Tully CJ (2009). Kompetenzerwerb im freiwilligen Engagement. Eine empirische Studie zum informellen Lernen im Jugendalter.

[CR9] Emrich E, Pierdzioch C (2014). Die Motive Ehrenamtlicher im Sport: Eine Lebenszyklusanalyse. Spectrum.

[CR10] Flick U (2010). Qualitative Sozialforschung. Eine Einführung.

[CR11] Flick U, Oelerich G, Otto H-U (2011). Das Episodische Interview. Empirische Forschung und Soziale Arbeit.

[CR12] Foucault M (2009). Hermeneutik des Subjekts. Vorlesungen am Collège de France.

[CR13] Fuhr T, Hof C, Rosenberg H (2018). Lernen im Lebenslauf als transformatives Lernen. Lernen im Lebenslauf. Theoretische Perspektiven und empirische Zugänge.

[CR14] Heckhausen J, Heckhausen H, Heckhausen H, Heckhausen J (2018). Motivation and action: introduction and overview. Motivation und action.

[CR15] Kade J, Nolda S (2015). Lernen im Kontext von Biografie und Lebenslauf. Zeitschrift für Weiterbildungsforschung-Report.

[CR16] Keil A, Nittel D, Seltrecht A (2013). Krankheit als biografischer Ausnahmezustand: Der objektive Faktor der Subjektivität. Krankheit: Lernen im Ausnahmezustand.

[CR17] Kelle U, Kluge S (2010). Vom Einzelfall zum Typus. Fallvergleich und Kontrastierung in der qualitativen Sozialforschung.

[CR18] Koller H-C (2018). Bildung anders denken. Einführung in die Theorie transformatorischer Bildungsprozesse.

[CR19] Koller H-C, Yacek D (2022). Bildung als Transformation? Zur Diskussion um die Theorie transformatorischer Bildungsprozesse. Bildung und Transformation. Zur Diskussion eines erziehungswissenschaftlichen Leitbegriffs.

[CR20] Kuckartz U (2018). Qualitative Inhaltsanalyse: Methoden, Praxis, Computerunterstützung.

[CR21] Lancee B, Radl J (2014). Volunteering over the life course. Social Forces.

[CR22] Maier-Gutheil C (2015). Lern- und Bildungsprozesse im Lebenslauf – Befunde empirischer Forschung und Perspektiven der Theorieentwicklung. Zeitschrift für Weiterbildungsforschung.

[CR23] Matthes J, Schütze F, Arbeitsgruppe Bielefelder Soziologen (1980). Zur Einführung: Alltagswissen, Interaktion und Gesellschaftliche Wirklichkeit (0). Alltagswissen, Interaktion, und gesellschaftliche Wirklichkeit. T.1, Symbolischer Interaktionismus und Ethnomethodologie.

[CR44] Matuschek I., Stanik T. (2021). Strukturelle Probleme, individuelle Probleme? Weiterbildung im Kontext von Beschäftigung(slosigkeit). DIE Zeitschrift für Erwachsenenbildung.

[CR24] McClelland DC, Koestner R, Weinberger J (1989). How do self-attributed and implicit motives differ?. Psychological Review.

[CR25] Mezirow J (2000). Learning as transformation: critical perspectives on a theory in progress.

[CR26] Nittel D, Nittel D, Seltrecht A (2013). Prozessuale Lerndimension: Instrumente zur Erschließung von Lernprozessen bei Patienten mit lebensbedrohlichen Erkrankungen. Krankheit: Lernen im Ausnahmezustand? Brustkrebs und Herzinfarkt aus interdisziplinärer Perspektive.

[CR27] Nittel D, Tippelt R, von Hippel A (2018). Biographietheoretische Ansätze in der Erwachsenenbildung. Handbuch Erwachsenenbildung/Weiterbildung.

[CR28] Nittel D, Seltrecht A (2013). Krankheit: Lernen im Ausnahmezustand? Brustkrebs und Herzinfarkt aus interdisziplinärer Perspektive.

[CR29] Oevermann U, Müller-Doohm S (1991). Genetischer Strukturalismus und das sozialwissenschaftliche Problem der Erklärung der Entstehung des Neuen. Jenseits der Utopie.

[CR30] Pekrun R, Huber M, Krause S (2018). Emotion, Lernen und Leistung. Bildung und Emotion.

[CR31] Rosenberg H, Hof C (2021). Stichwort. Kritische Lebensereignisse. Weiter bilden. DIE Zeitschrift für Erwachsenenbildung.

[CR34] Rüber IA, Janmaat JG (2021). Does participation in adult education increase volunteering? An analysis of British longitudinal data. Adult Education Quarterly.

[CR33] Rüber IA, Rees S-L, Schmidt-Hertha B (2018). Lifelong Learning—lifelong returns? A new theoretical framework for the analysis of civic returns on adult learning. International Review of Education.

[CR32] Ryan RM, Deci EL (2017). Self-determination theory: Basic psychological needs in motivation, development, and wellness.

[CR35] Sautermeister J (2021). Aufbrechende Sinnfragen: Kritische Lebensereignisse als „Lernchancen“ im höheren Lebensalter. Weiter bilden. DIE Zeitschrift für Erwachsenenbildung.

[CR36] Schütz A (1972). Gesammelte Aufsätze II. Studien zur soziologischen Theorie.

[CR37] Simonson J, Vogel C, Tesch-Römer C (2017). Freiwilliges Engagement in Deutschland: Der Deutsche Freiwilligensurvey 2014.

[CR38] Son J, Wilson J (2017). Education, perceived control, and volunteering. Sociological Forum.

[CR39] Sprung A, Kukovetz B (2018). Refugees welcome? Active Citizenship und politische Bildungsprozesse durch freiwilliges Engagement. Zeitschrift für Weiterbildungsforschung.

[CR40] Sung P, Chia A, Chan A, Malhotra R (2023). Reciprocal relationship between lifelong learning and volunteering among older adults. The Journals of Gerontology: Series B.

[CR42] Trautwein C, Bosse E (2017). The first year in higher education-critical requirements from the student perspective. Higher education.

[CR43] Wilson J (2012). Volunteerism research: A review essay. Nonprofit and voluntary sector quarterly.

